# Hepatitis A Associated with Semidried Tomatoes, France, 2010

**DOI:** 10.3201/eid1703.101479

**Published:** 2011-03

**Authors:** Céline Gallot, Lise Grout, Anne-Marie Roque-Afonso, Elisabeth Couturier, Paloma Carrillo-Santisteve, Jérôme Pouey, Marie-José Letort, Stéphanie Hoppe, Pascal Capdepon, Sylvie Saint-Martin, Henriette De Valk, Véronique Vaillant

**Affiliations:** Author affiliations: French Institute for Public Health Surveillance, Saint Maurice, France (C. Gallot, E. Couturier, P. Carrillo-Santisteve, M.-J. Letort, H. De Valk, V. Vaillant);; Midi-Pyrénées Interregional Epidemiology Unit, Toulouse, France (L. Grout, J. Pouey);; Hôpital Paul Brousse, Villejuif, France (A.-M. Roque-Afonso);; Aquitaine Interregional Epidemiology Unit, Bordeaux, France (S. Hoppe);; Midi-Pyrénées Health Regional Agency, Tarbes, France (P. Capdebon, S. Saint-Martin)

**Keywords:** Hepatitis A, viruses, hepatitis viruses, semidried tomatoes, France, letter, *Suggested citation for this article:* Gallot C, Grout L, Roque-Afonso A-M, Couturier E, Carrillo-Santisteve P, Pouey J, et al. Hepatitis A associated with semidried tomatoes, France, 2010 [letter]. Emerg Infect Dis [serial on the Internet]. 2011 Mar [*date cited*]. http://dx.doi.org/10.3201/eid1703.101479

**To the Editor:** In January 2010, two clusters of nontraveler-associated hepatitis A were reported in 3 districts of southwestern France. A single IB strain of hepatitis A virus (HAV) was isolated (FR-2010-LOUR, GenBank accession no. GU646039)*.* We conducted an investigation to describe the outbreak, identify the vehicle of transmission and source of infection, and propose appropriate control measures.

Cases were identified through mandatory notification or through the National Reference Centre for HAV. A total of 59 cases were identified: 49 confirmed cases (resident of France and infected with the outbreak strain) and 10 probable cases (resident of southwestern France and with a locally acquired infection positive for HAV immunoglobulin M against HAV with onset during November 1, 2009–February 28, 2010). Twelve (20%) persons were secondary case-patients (symptom onset 2–6 weeks after contact with a case-patient).

Twenty-eight (47.5%) case-patients were hospitalized, and all recovered. Case-patients were 7–54 years of age (median 31.5 years). The male:female ratio was 1.2:1. Cases were scattered throughout France, with 1 cluster each in Lot and Hautes-Pyrénées districts. Case-patients reported symptom onset during November 20, 2009–February 17, 2010 (Figure), with peaks during December 20, 2009–January 2, 2010, and January 24–30, 2010. The epidemiologic curve suggested a persistent common source of contamination, followed by person-to-person transmission. Of the 47 persons with nonsecondary cases (primary cases and cases that were not able to be classified), 27 (57%) reported having eaten in a sandwich shop. Twenty-four (51%) reported eating semidried tomatoes, 20 of 566m reported purchasing semidried tomatoes in 1 of 3 different sandwich shop chains.

**Figure:**
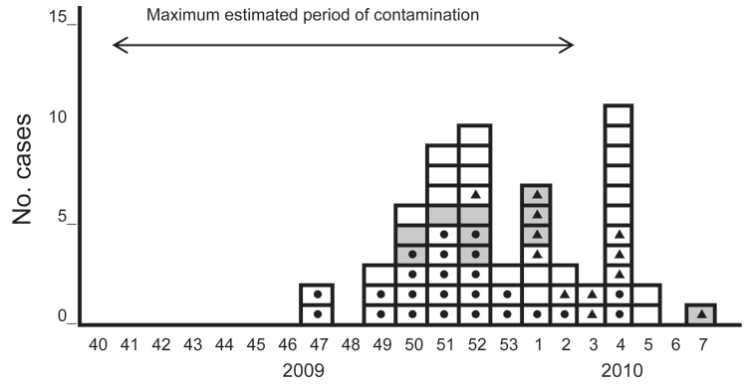
Weekly distribution of hepatitis A cases, by date of onset, France, November 2009–February 2010. White boxes indicate confirmed cases; gray boxes indicate probable cases; dots indicate patient consumed semidried tomatoes; triangles indicate secondary cases. French calendar designates 53 weeks in 2009 (week 53, December 28, 2009–January 3, 2010).

We conducted a case–control study of 30 nonsecondary case-patients with symptom onset during November 22, 2010–January 9, 2010; 109 controls (15–60 years of age living in the same district as case-patients and without histories of HAV infection or hepatitis A vaccination) were selected by random digit dialing. Exposures occurring 2–6 weeks before illness onset (case-patients) and before interview (controls) were recorded by telephone by using a standardized questionnaire. Logistic regression was performed (Stata 9.2; StataCorp LP, College Station, TX, USA); p<0.05 was considered statistically significant. Case-patients were more likely than controls to have eaten sandwiches or salads from a sandwich shop (age-adjusted odds ratio 29.1, 95% confidence interval 9.7–87.0) and to have eaten semidried tomatoes (age-adjusted odds ratio 8.5, 95% confidence interval 4.4–30.2).

HAV genotyping was performed as described (*1*). The epidemic strain FR-2010-LOUR was genotype IB. No identical strain was found in the National Reference Centre for HAV sequence database, even though IB strains represented one third of routinely isolated strains. The strain clustered significantly with sequences from patients returning from Turkey.

Trace-back investigations identified a supplier in France that imported frozen semidried tomatoes from Turkey and supplied the 3 sandwich shop chains. In France, the frozen semidried tomatoes were defrosted and processed with oil and herbs before distribution. No heat treatment, disinfection, or washing was conducted after defrosting. The period of distribution of 1 batch matched the estimated period of contamination of nonsecondary cases. This batch was no longer available at the supplier or at the sandwich shops for virologic analysis or for recall.

Our results suggest that this nationwide hepatitis A outbreak was associated with eating 1 batch of semidried tomatoes imported from Turkey and processed in France. Infected food handlers are the most frequently documented source of contamination by HAV of food items, but food also can be contaminated by contact of products or machinery with contaminated water (*2*). Therefore, the tomatoes may have been contaminated during processing by the supplier in France, during production in Turkey, or during growing. Fecal contamination of foods that are not subsequently cooked is a potential source of HAV, and the virus remains infectious for long periods, even after freezing (*3*). Various fresh or frozen produce have been associated with hepatitis A outbreaks (*4*,*5*).

Recently, 3 other hepatitis A outbreaks were associated with eating semidried tomatoes: in Australia in May and November 2009 and in the Netherlands in 2010 (*6*–*9*). All 4 outbreaks were caused by highly similar IB strains, although the French outbreak strain differed by 2 nt from the Australian strain (based on a 300-nt fragment of the VP1–2A part of the genome), and by 3 nt from the Dutch strain (on the basis of a 430-nt fragment) (*8*).

During the past decade, hepatitis A incidence has decreased considerably in western Europe (*10*). The low incidence and low vaccine coverage have led to a high proportion of susceptible persons, which creates the potential for extended hepatitis A outbreaks if contaminated products are widely distributed. Imported products from regions with high endemicity are widely distributed throughout Europe, and some have a long shelf life, (especially if frozen). Semidried tomatoes should be considered a potential vehicle of transmission in foodborne outbreaks of HAV.
